# The association between magnesium levels and gout: evidence from Mendelian randomization, a Chinese cross-sectional study, and NHANES analysis

**DOI:** 10.3389/fnut.2025.1688095

**Published:** 2025-11-25

**Authors:** Congcong Jiao, Yang Shao, Yuxin Zhao, Ruichao Feng, Xiangfei Cui, Junjun Luan, Xiangnan Hao, Cong Ma, Haoshen Feng, Xu Yang, Hua Zhou

**Affiliations:** 1 Department of Nephrology, Shengjing Hospital of China Medical University, Shenyang, China; 2 Department of Rehabilitation Medicine, First Hospital of China Medical University, Shenyang, China; 3 Department of Nephrology, The Affiliated First Hospital of Jinzhou Medical University, Jinzhou, China; 4 Department of Pulmonary and Critical Care Medicine, Shengjing Hospital of China Medical University, Shenyang, China

**Keywords:** gout, magnesium, Mendelian randomization, cross-sectional study, NHANES

## Abstract

**Introduction:**

Although the roles of micronutrients in human health are widely acknowledged, their specific associations with gout remain inadequately explored. This study integrates evidence from Mendelian randomization (MR), Chinese cross-sectional, and NHANES analyses to comprehensively investigate.

**Methods:**

The MR analysis was used to evaluate the potential causal associations between 15 trace elements (copper, calcium, iron, magnesium, potassium, selenium, zinc, carotenoids, folate, vitamin A, vitamin B12, vitamin B6, vitamin C, vitamin D, and vitamin E) and gout risk from the FinnGen database (*n* = 327,457). Significant findings were validated via logistic regression in Chinese clinical data (*n* = 4,359) and NHANES 2011-2018 data (*n* = 13,902).

**Results:**

Univariable MR identified calcium, magnesium, and vitamin B6 as associated with gout. Multivariable MR indicated that only higher magnesium levels causally reduced gout risk (OR = 0.630, 95% CI: 0.400-0.992, *p* = 0.046). Consistently, high serum magnesium (Q4) was associated with lower gout risk in the Chinese clinical data (OR = 0.546, 95% CI: 0.319–0.933, *p* = 0.027) versus the lowest quartile (Q1). NHANES analysis confirmed that higher dietary magnesium intake lowered gout risk (OR = 0.738, 95% CI: 0.550–0.989, *p* = 0.049). Additionally, the restricted cubic spline (RCS) found that the OR began below 1 when the dietary magnesium intake exceeded 0.27 g/day.

**Discussion:**

This multifaceted study provides novel evidence supporting a protective role of magnesium against gout. The underlying mechanism may involve magnesium’s influence on uric acid or its anti-inflammatory effects. These hypotheses need to be clarified by further experimental and clinical studies.

## 1 Introduction

Patients with gout, a chronic condition caused by monosodium urate crystal deposition, typically present with acute inflammatory monoarthritis, often affecting the lower limb joints (1). Key risk factors include elevated uric acid production (e.g., from intake of high-purine foods), diminished uric acid excretion (e.g., from renal dysfunction), and inflammatory responses (2). The global incidence of gout markedly increased between 1990 and 2019, particularly in high-income regions of North America and East Asia (3). The number of people with gout worldwide reached 55.8 million in 2020 and is projected to continue to increase by 2050. Therefore, an urgent need exists to strengthen health education and promote improvements in diet and lifestyle to alleviate the burden of gout (4).

In recent years, accumulating evidence has established that various micronutrients (minerals and vitamins) are significantly associated with both serum urate homeostasis and gout pathogenesis, and have critical roles in uric acid metabolism. Among essential mineral elements, novel copper- and zinc-based compounds have shown potent dual inhibition of xanthine oxidase and xanthine dehydrogenase enzymatic activities, thus resulting in urate-lowering effects (5). Dietary potassium, particularly from fruit sources, has been suggested to exert urate-lowering effects (6). Epidemiological investigations have revealed inverse associations of magnesium and iron with gout risk (7), whereas serum calcium concentrations have shown positive correlations with both hyperuricemia and gout incidence (8). Furthermore, blood selenium levels have been found to have significant positive correlations with serum urate concentrations and hyperuricemia (HUA) prevalence (9); consequently, selenium might be a novel risk factor for gout development. Among the vitamins, serum retinol (a vitamin A metabolite) is positively correlated with uric acid levels (10), whereas vitamin B12 and folate intake are inversely associated with serum urate and gout risk, respectively (8). Vitamin C intake is negatively correlated with serum uric acid levels (10), and vitamin C supplementation is further associated with a modest decrease in gout risk (11). Notably, individuals with vitamin D insufficiency or deficiency have significantly higher serum uric acid levels than those with normal vitamin D status (12).

These findings highlight the potential importance of micronutrients in urate metabolism and gout risk management, yet comprehensive systematic analyses of the relationship between micronutrients and gout incidence remain limited. Therefore, we conducted a Mendelian randomization (MR) analysis to screen micronutrients, and subsequently used clinical data and the NHANES database to explore associations between micronutrients and gout.

## 2 Materials and methods

### 2.1 Mendelian randomization analysis

#### 2.1.1 Date source

**Figure 1:**
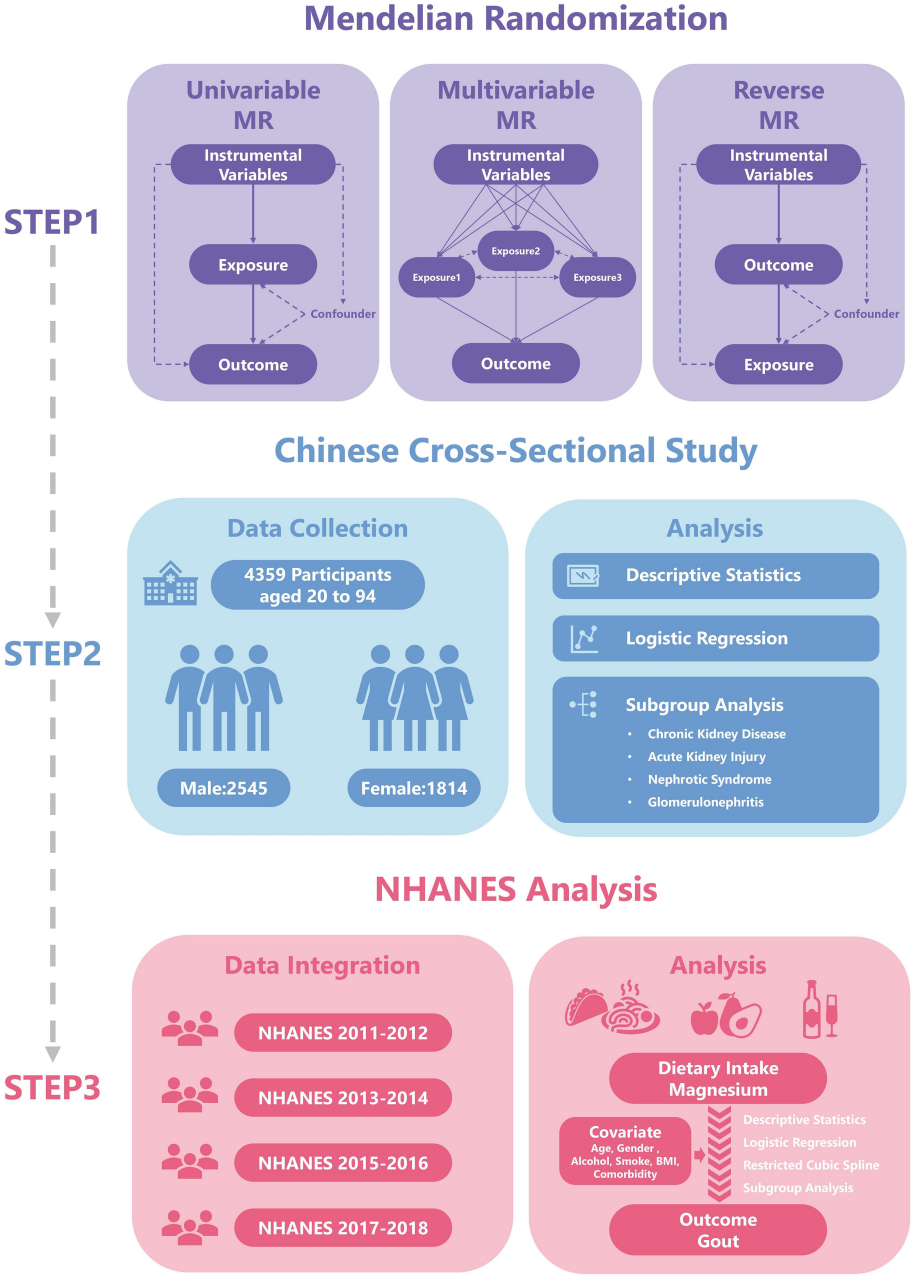
The flowchart has been revised. Specifically, the term “Two-sample MR” has been updated to “Univariable MR”, and the title of the second part has been changed to “Chinese Cross-Sectional Study”.

The analytical workflow of this study is presented in Figure 1. On the basis of previous studies, we collected genetic data for 15 trace elements from the Genome-wide Association Study (GWAS) database. These 15 micronutrients comprised vitamin A (ukb-b-9596, European); vitamin B12 (ukb-b-19524, European); vitamin B6 (ukb-b-7864, European); vitamin C (ukb-b-19390, European); vitamin D (ukb-b-18593, European); vitamin E (ukb-b-6888, European); zinc (ieu-a-1079, European); copper (ieu-a-1073, European); calcium (ukb-b-8951, European); carotene (ukb-b-16202, European); folate (ukb-b-11349, European); iron (ukb-b-20447, European); magnesium (ukb-b-7372, European); potassium (ukb-b-17881, European); selenium (ieu-a-1077, European) (13). The outcome of interest in this study was gout. Relevant data were downloaded from the FinnGen database^1^ of European origin. This ancestry matching effectively avoids potential bias caused by population stratification.

#### 2.1.2 Instrumental variable screening

In this study, the TwoSampleMR package in R was used to screen single-nucleotide polymorphisms (SNPs), thus ensuring a strong association between instrumental variables (IVs) and the exposure (*p* < 5 × 10^−6^). To enhance the independence of selected SNPs, we set the clumping parameter to clump = TRUE. Using the parameter kb = 5,000 to define clustering window; within this window, SNPs with pairwise correlations exceeding the predefined threshold (*r*^2^ = 0.001) were considered correlated. Finally, we calculated and reported the F-statistic for each SNP and the mean F-statistic for each set of genetic instruments to confirm they are strong instruments (*F*-value >10), mitigating concerns about weak instrument bias.

#### 2.1.3 Analysis process

We used the VariantAnnotation, gwasglue, and TwoSampleMR packages to investigate the causal relationships between exposure factors and outcome variables. We applied methods including MR Egger, weighted median, inverse variance weighted (IVW), simple mode, and weighted mode to assess the causal relationships between multiple micronutrients and gout. Furthermore, we used reverse MR to explore the potential effects of the disease on exposure factors and to investigate the direction of causal relationships. Finally, we performed multivariable MR with the TwoSampleMR and MendelianRandomization packages in R, thereby accounting for the effects of multiple exposure factors.

#### 2.1.4 Sensitivity analysis

We identified potential biases and confounding factors through the leave-one-out method, heterogeneity test, and horizontal pleiotropy test. Leave-one-out analysis was conducted to evaluate the effect of each SNP on the overall effect by excluding each SNP one by one. The heterogeneity test was used to evaluate differences in effects associated with genetic variations by examining whether the effects of multiple IVs were consistent, according to the Q-statistic (MR Egger method) and I^2^ ( $I^{2}=\frac{Q-\mathrm{df}}{Q}\times 100\%$ ). The horizontal pleiotropy test used the MR-PRESSO and the MR Egger intercept method to examine whether the IVs directly affect the results through exposure factors. Finally, we visualized the results by generating funnel charts, leave-one-out forest plots, and scatter plots.

### 2.2 Clinical data validation

#### 2.2.1 Research design and sample size calculation

This study used a cross-sectional study design to obtain clinical data. According to literature reports (14), the overall standardized prevalence of gout in Chinese adults is 3.2% (*p* = 0.032). We set the error range (d) to 0.01 and the confidence level (*α* = 0.05) to 95% (Z = 1.96). According to the formula $N=\frac{Z_{\alpha /2}^{2}\times P(1-P)}{d^{2}}$ . The minimum required sample size was calculated to be 1,190 people. The sample size in this study exceeded this estimate.

#### 2.2.2 Data collection and processing

The research sample comprised patients who received inpatient treatment in a nephrology department (January 1, 2023 – December 31, 2023). According to prevailing international guidelines, applying the 2015 American College of Rheumatology/European League Against Rheumatism gout classification criteria (15), whereby a score of ≥ 8 points indicates definite gout, or by confirming a documented prior history of gout established. Basic data collection was performed with a dual independent input system, including the following core variables: demographic characteristics (age and sex); laboratory test findings (serum magnesium concentration in mg/dL, reference ranges: 1.63–2.80 mg/dL); diagnostic information (diagnosed gout); health related behaviors [smoking history, alcohol consumption history, and body mass index (BMI)] and other clinical diagnoses (chronic kidney disease [CKD], acute kidney injury [AKI], nephrotic syndrome [NS], and glomerulonephritis [GN]). Since all clinical data in this study were derived from nephrology patients. We estimated glomerular filtration rate (eGFR, mL/min/1.73 m^2^) using the Chronic Kidney Disease Epidemiology Collaboration (CKD-EPI) equation (16) and incorporated eGFR into the study. The data processing flow strictly followed clinical research standards, and data cleaning and analysis were conducted in R.

### 2.3 NHANES analysis

#### 2.3.1 Study population

We downloaded health and nutrition data from the NHANES database spanning 2011 to 2018, including demographic data (sex and age), weight data (WTINT2YR, WTMEC2YR, SDMVPSU, and SDMVSTRA), dietary intake data (Magnesium, in g), disease status (gout), and other health-related conditions (smoking history, alcohol consumption history, BMI, high blood pressure, high cholesterol, and diabetes).

#### 2.3.2 Data organization

We used 24-h dietary recall interviews from the NHANES database to calculate the intake of trace elements. The first interview was conducted at the Mobile Examination Center, and the second interview was conducted via telephone follow-up after 3–10 days. On the basis of the results of these two interviews, we calculated the average of the two intake levels. If data from one interview were missing, we used the results from the other interview. The outcome was determined according to participants’ self-reported history of gout in the NHANES database. Finally, the mice package in R was used to filter and fill in the gaps in the NHANES dataset.

### 2.4 Statistical analysis

This study performed MR analysis using the R language (Version 4.5.1), including univariate MR analysis, reverse MR analysis, and multifactorial MR analysis (Supplementary material 1). And we constructed a logistic regression model in R to explore the relationship between exposure factors and gout. We first constructed a single-factor regression model (model 1), considering only the direct effects of exposure factors on gout, as a preliminary analysis. On the basis of the single-factor model, we extended the multiple regression model (model 2) by adding sex and age as covariates. We also constructed a more comprehensive regression model (model 3), which added other factors potentially affecting the outcome variables, including BMI, alcohol consumption, smoking status, and comorbidities. We used the Forestplotter package to create a forest plot for subgroup analysis, displaying odds ratios (OR), 95% confidence intervals (CIs), and their significance levels. In addition, we explored the nonlinear relationship between dietary intake of magnesium and gout by using a restricted cubic spline (RCS) to analyze the effects of dietary magnesium intake on gout. Both logistic regression and restricted cubic spline models fully accounted for the complex multistage survey design of NHANES by utilizing weighted data. This study employed multiple imputation using the mice package to handle missing data, with all analyses conducted based on the combined effective sample size. All reported *p*-values represent two-tailed tests, and the threshold for statistical significance is explicitly stated (*p* < 0.05).

## 3 Results

### 3.1 Results of Mendelian randomization analysis

#### 3.1.1 Univariable Mendelian randomization analysis

**Figure 2:**
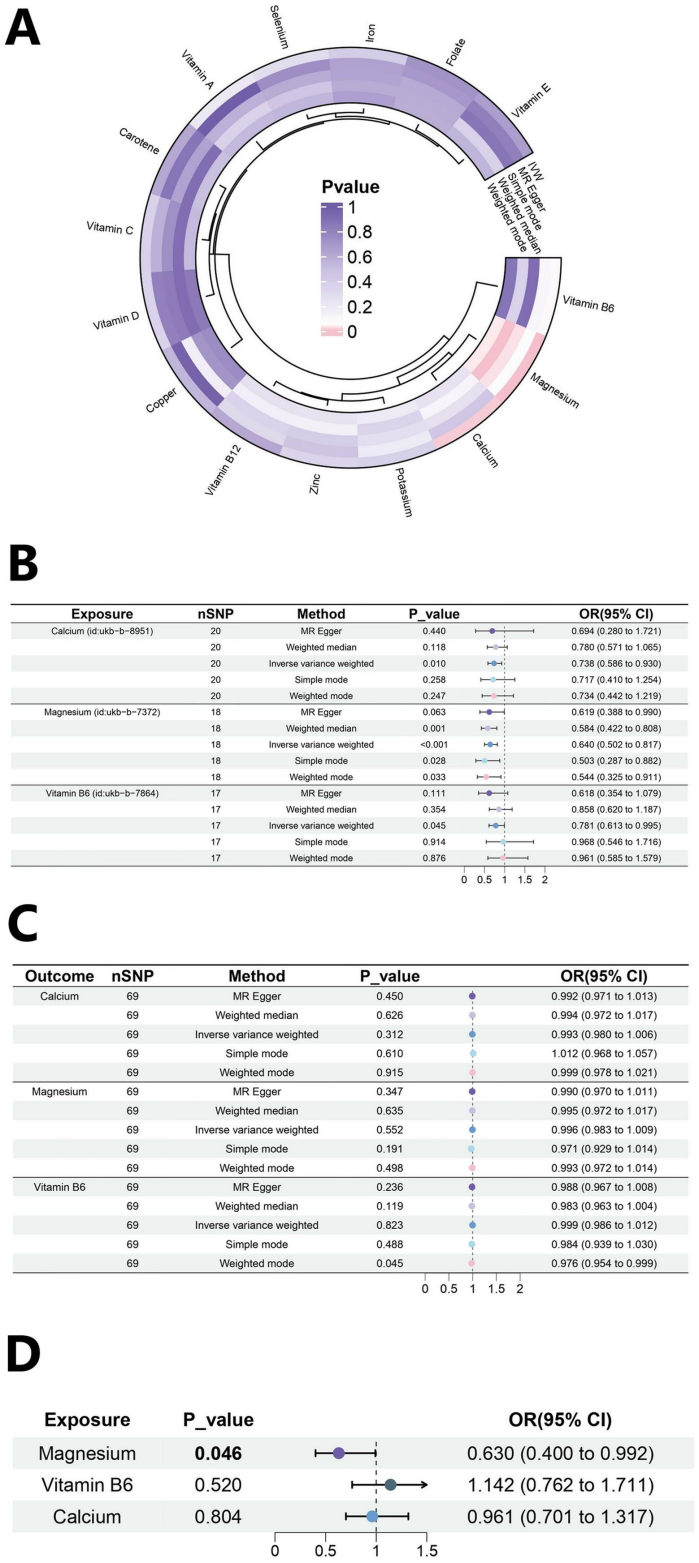
Mendelian randomization analysis results. **(A)** Heat map of univariate MR analysis for 15 micronutrients. **(B)** Forest plot of MR analysis for calcium, magnesium and Vitamin B6. **(C)** Forest plot of reverse MR analysis. **(D)** Forest plot of multivariate MR analysis.

The outcome of this study was gout, which included 12,342 gout cases and 315,115 controls. The SNPs with bias were excluded by the MR-PRESSO test. A total of 171 SNPs were included in this study, and their *F* values were all >10 (Supplementary Table 1). In the MR results between 15 trace elements and gout (Supplementary Table 2; Supplementary Figure 1), calcium, magnesium, and vitamin B6 were screened out (Figure 2A). The IVW analysis results indicated *p* < 0.05, and the results of other methods were consistent with the IVW method direction. Specifically, the OR for calcium was 0.738 (95% CI: 0.586–0.930, *p* = 0.010), the OR for magnesium was 0.640 (95% CI: 0.502–0.817, *p* < 0.001), and the OR for vitamin B6 was 0.781 (95% CI: 0.613–0.995, *p* = 0.045) (Figure 2B). These results suggested that calcium, magnesium, and vitamin B6 might be protective factors against gout.

#### 3.1.2 Sensitivity analysis

**Figure 3:**
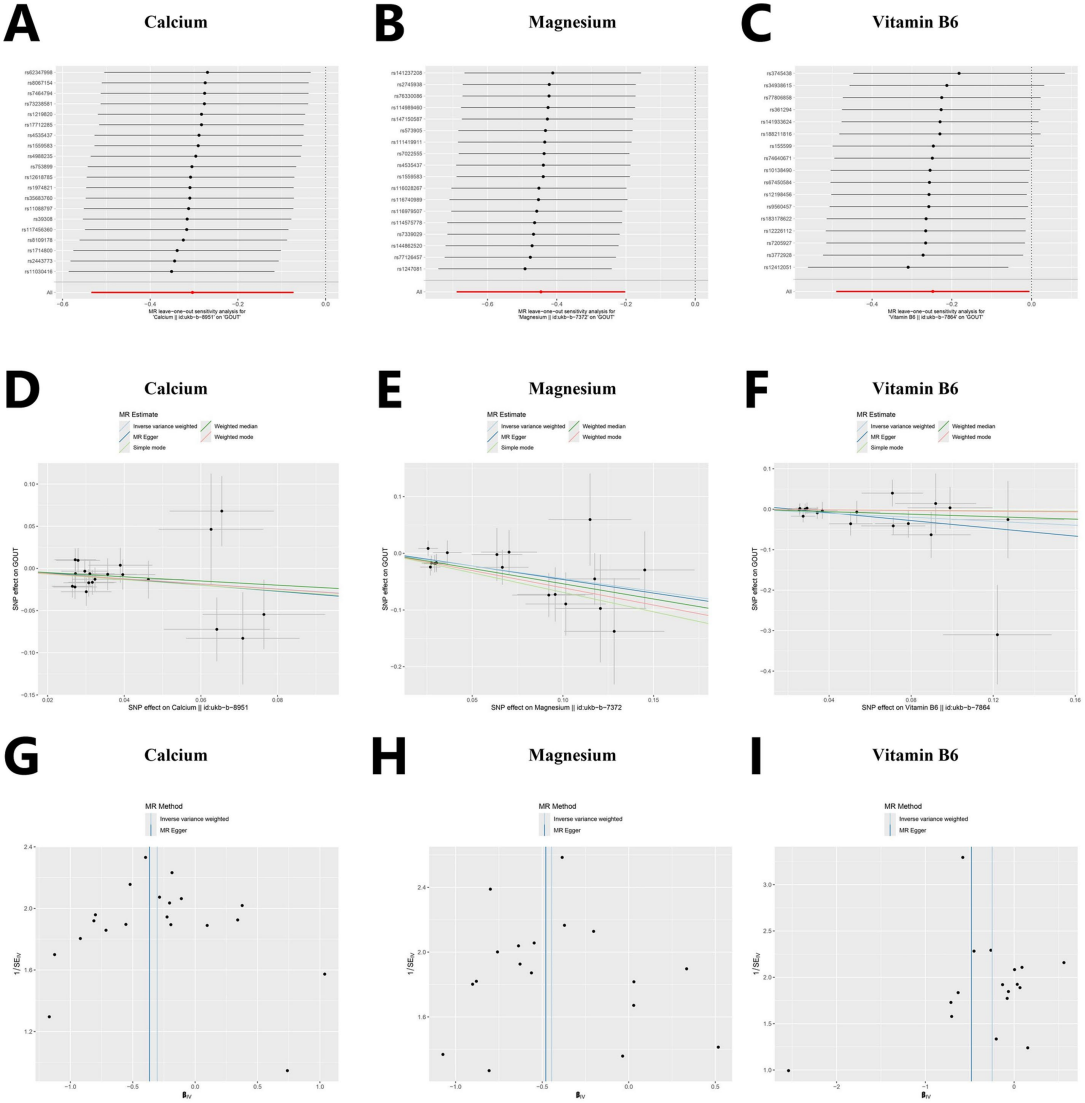
Sensitivity analysis for Mendelian randomization. **(A–C)** Forest plots of leave-one-out sensitivity analysis. **(D–F)** Scatter plots of five analytical methods. **(G–I)** Funnel plots of heterogeneity analysis.

The results of the sensitivity analysis of Calcium, magnesium, and vitamin B6 are as follows. According to the sensitivity analysis with the leave-one-out method (Figures 3A–C), the overall results did not significantly change after the exclusion of instrumental variables one by one. A scatter plot visualizing the relationships between exposures and the outcome (Figures 3D–F). The roughly symmetrical funnel plot (Figures 3G–I) indicated no significant bias and supported the reliability of the results. In the heterogeneity test, no significant difference was observed (*p* > 0.05, I^2^ = 0.00%), and the results of the horizontal pleiotropy analysis were also not statistically significant (*p* > 0.05), thus further indicating the robustness of the results of this study. The results of other micronutrients are detailed in Supplementary Tables 3–5 and Supplementary Figures 2-4.

#### 3.1.3 Reverse Mendelian randomization analysis

Reverse MR analysis is used primarily to evaluate whether a reverse causal relationship exists between an exposure and outcome (Supplementary Table 6). The analysis demonstrated that gout did not significantly (*p* > 0.05) affect the levels of calcium (OR = 0.993, 95% CI: 0.980–1.006), magnesium (OR = 0.996, 95% CI: 0.983–1.009), and vitamin B6 (OR = 0.999, 95% CI: 0.986–1.012) (Figure 2C). The sensitivity analysis values are as follows: calcium (Heterogeneity *p* = 0.290; Pleiotropy *p* = 0.868), magnesium (Heterogeneity *p* = 0.375; Pleiotropy *p* = 0.463), and vitamin B6 (Heterogeneity *p* = 0.641; Pleiotropy *p* = 0.180). This result indicates the direction of causality between the exposure factors and the outcome.

#### 3.1.4 Multivariable Mendelian randomization analysis

A multivariable MR analysis was performed to investigate the collective effects of three mineral exposures—calcium, magnesium, and vitamin B6—on gout risk. We extracted IVs from each exposure (*p* < 5 × 10^−6^, kb = 5,000 kb, r^2^ = 0.001), then merged these exposure-specific IVs for multivariate analysis. The strength of IVs was assessed using conditional F-statistics, which were 12.68 for calcium, 14.25 for magnesium, and 11.76 for vitamin B6, all exceeding the conventional threshold of 10. The proportion of variance explained by each exposure after adjusting for others, denoted as conditional R^2^, was 0.884 for calcium, 0.945 for magnesium, and 0.917 for vitamin B6. In the multivariable MR, calcium and vitamin B6 were not significantly associated with gout. In contrast, magnesium remained a significant protective factor (OR = 0.630, 95% CI: 0.400–0.992, *p* = 0.046), as illustrated in Figure 2D. Diagnostics for pleiotropy and heterogeneity were conducted using MV-IVW and MV-Egger regression. The Cochran’s Q statistic was 39.582 (*p* = 0.877), suggesting no substantial heterogeneity, and the MR-Egger intercept was −0.0008 (*p* = 0.909), indicating no evidence of directional pleiotropy.

### 3.2 Clinical data validation

#### 3.2.1 Baseline characteristics of the survey population

**Figure 4:**
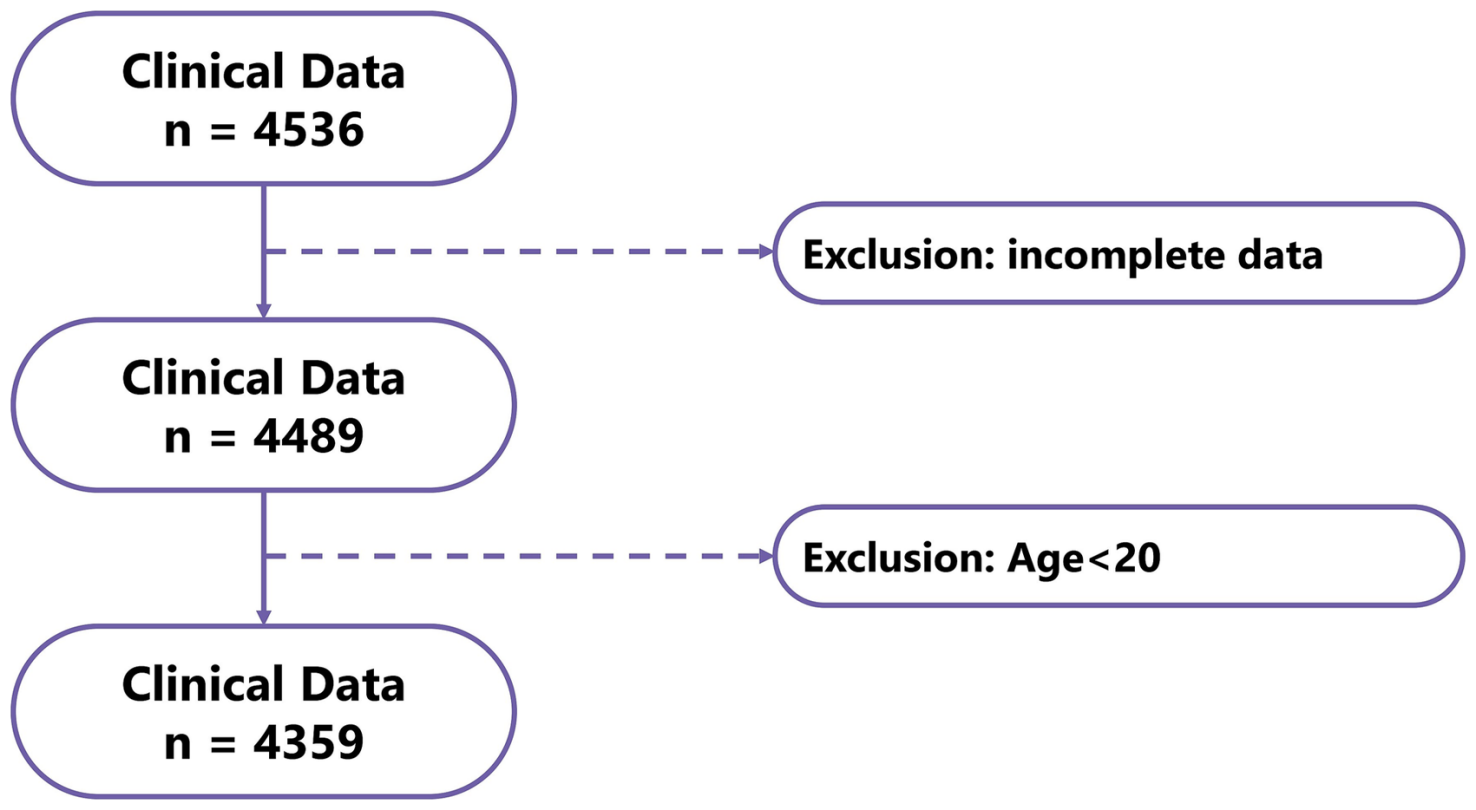
Flowchart of clinical data sample screening.

**Figure 5:**
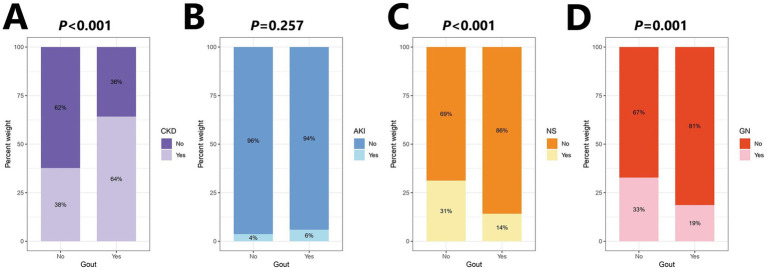
Percentage stacked bar chart of kidney disease distribution. **(A)** Chronic kidney disease (CKD). **(B)** Acute kidney injury (AKI). **(C)** Nephrotic syndrome (NS). **(D)** Glomerular nephritis (GN).

Data for 4,536 patients were collected in this study, and those for 4,359 patients (Control: 4225; Case: 134) who met the criteria were included in the analysis (Figure 4). This study was approved by the Institutional Review Board of Shengjing Hospital of China Medical University. The clinical data indicated lower serum magnesium in the gout group than the control group, although no significant difference was observed between groups (*p* = 0.539). Moreover, we observed significant differences in factors such as age, sex, BMI, alcohol consumption, smoking habits, and eGFR between the gout and control groups (Table 1). Because the data all came from nephrology patients, we also explored the relationship between gout and kidney disease. The results indicated significant differences in the proportions of CKD (64%), NS (14%), and GN (19%) between the gout group and the control group (Figure 5).

**Table 1:** Baseline information of clinical data.

Characteristics	Overall	No	Yes	*P*-value
*N*	4,359	4,225	134	
Serum magnesium	2.141 (0.348)	2.141 (0.349)	2.122 (0.317)	0.539
Age	53.867 (14.428)	54.103 (14.405)	46.440 (13.155)	**<0.001**
BMI	24.883 (4.059)	24.815 (4.022)	27.043 (4.593)	**<0.001**
eGFR	60.583 (40.751)	61.080 (40.787)	44.901 (36.382)	**<0.001**
Gender (%)				
Female	1814 (41.6%)	1804 (42.7%)	10 (7.5%)	**<0.001**
Male	2,545 (58.4%)	2,421 (57.3%)	124 (92.5%)	
Alcohol (%)				
No	3,270 (75.0%)	3,182 (75.3%)	88 (65.7%)	**0.015**
Yes	1,089 (25.0%)	1,043 (24.7%)	46 (34.3%)	
Smoke (%)				
No	2,783 (63.8%)	2,711 (64.2%)	72 (53.7%)	**0.017**
Yes	1,576 (36.2%)	1,514 (35.8%)	62 (46.3%)	

The bold values indicate statistical significance (*P* < 0.05).

#### 3.2.2 Logistic regression analysis of serum magnesium

According to the serum magnesium quartile (Q1 < 1.920 mg/dL, Q2 = 1.920 to <2.114 mg/dL, Q3 = 2.114 to <2.309 mg/dL, Q4 ≥ 2.309 mg/dL), we divided participants into four groups: Q1 to Q4. The distribution of eGFR differed significantly across serum magnesium quartiles (*p* < 0.001), justifying the inclusion of eGFR as a covariate in the multivariable logistic regression model to control for its confounding effect (Supplementary Figure 5). In a logistic regression model considering sex, age, BMI, smoking history, alcohol consumption, CKD, AKI, NS, GN, and eGFR (Table 2; Supplementary Table 7). We found the Q4 group may have a potentially protective effect on gout than the Q1 group (OR = 0.546, 95% CI: 0.319–0.933, *p* = 0.027).

**Table 2:** The logistic regression model of exposure factor in clinical data.

Serum magnesium	Gout		Model 1				Model 2				Model 3			
	Yes	No	*OR*	95%*CI* low	95%*CI* up	*P*-value	*OR*	95%*CI* low	95%*CI* up	*P*-value	*OR*	95%*CI* low	95%*CI* up	*P*-value
Q1: <1.920 mg/dL	38	1,119	Reference											
Q2: 1.920 to <2.114 mg/dL	29	1,136	0.752	0.460	1.227	0.254	0.804	0.489	1.320	0.388	0.799	0.481	1.327	0.386
Q3: 2.114 to <2.309 mg/dL	39	973	1.180	0.749	1.860	0.475	1.293	0.815	2.054	0.275	1.062	0.659	1.712	0.804
Q4: ≥2.309 mg/dL	28	997	0.827	0.504	1.357	0.453	0.985	0.596	1.629	0.953	0.546	0.319	0.933	**0.027**

The bold values indicate statistical significance (*P* < 0.05).

#### 3.2.3 Subgroup analysis of the link between serum magnesium and gout

**Figure 6:**
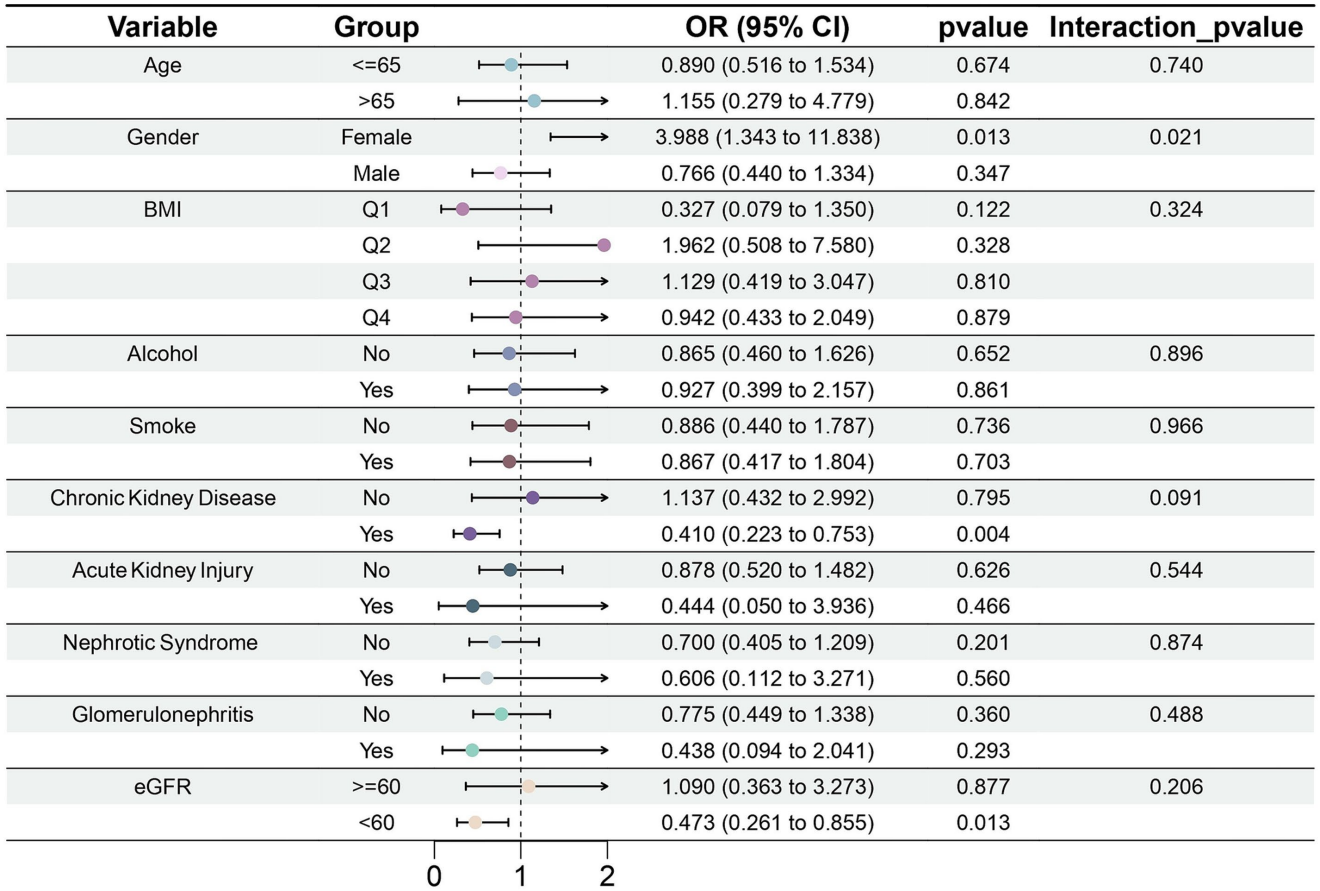
Forest plot of subgroup analysis of the effect of serum magnesium on gout.

In the CKD subgroup, serum magnesium concentration was significantly negatively correlated with gout risk (OR = 0.410, 95% CI: 0.223–0.753, *p* = 0.004) (Figure 6). This outcome was consistent with eGFR. We found that in the subgroup with eGFR <60 mL/min/1.73 m^2^, magnesium may also be a protective factor against gout (OR = 0.473, 95% CI: 0.261–0.855, *p* = 0.013). However, among female participants, greater serum magnesium concentrations were associated with a higher risk of gout (OR = 3.998, 95% CI: 1.343–11.838, *p* = 0.013). It is critical to interpret this finding with extreme caution due to the very limited number of female gout cases in our study (Control: 1804; Case: 10), which considerably undermines the statistical reliability. This caution is further warranted by the significant interaction effect observed between sex and serum magnesium (*p* for interaction = 0.021). Moreover, analysis conducted exclusively in males, which comprised the majority of cases, showed no significant association (OR = 0.766, 95% CI: 0.440–1.334, *p* = 0.347). Collectively, given the instability of the female subgroup estimate and the null finding in males. The result should currently be considered exploratory, pending validation in large-scale, prospective cohorts.

### 3.3 NHANES analysis

#### 3.3.1 Baseline characteristics of the study population

**Figure 7:**
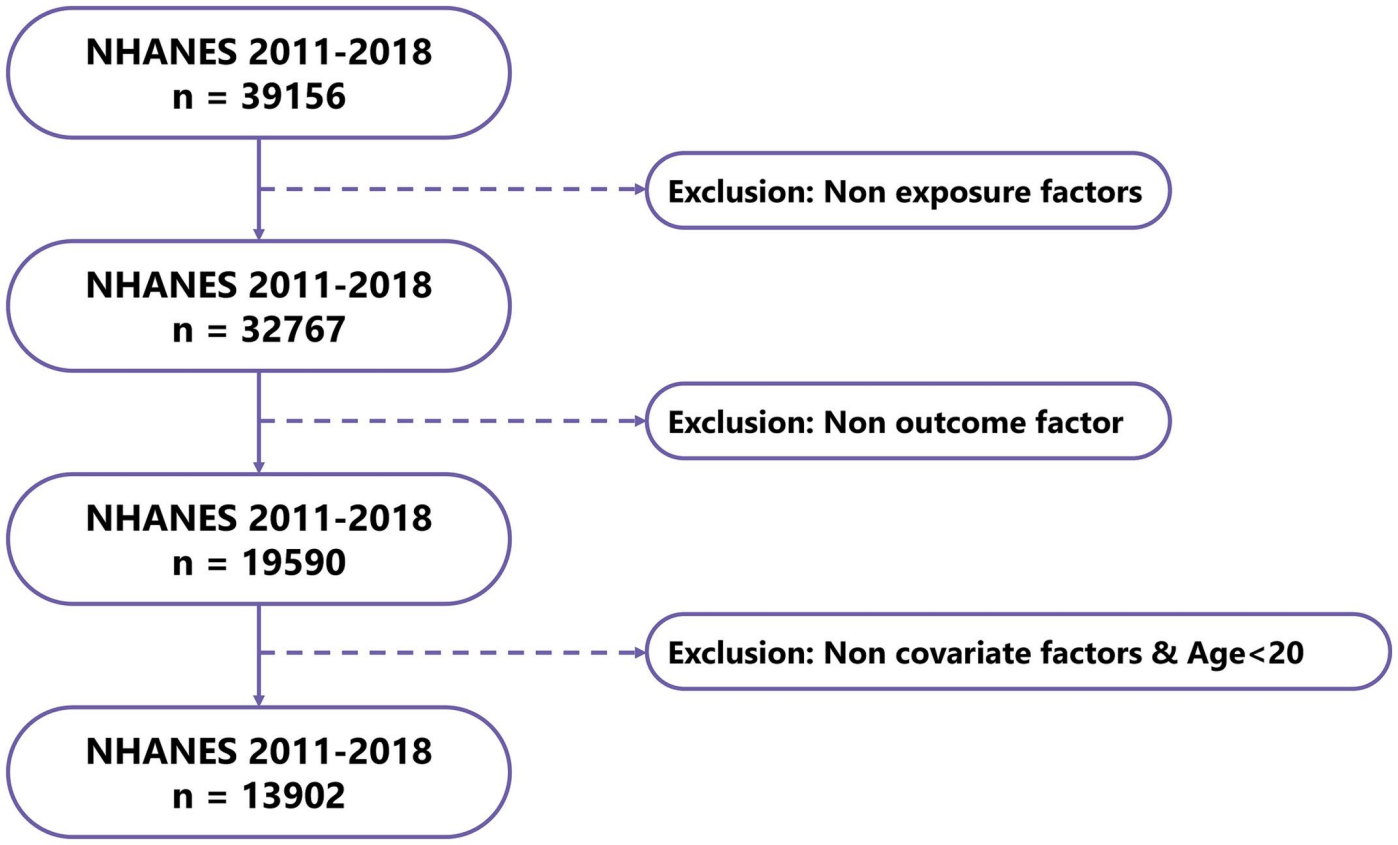
Flowchart of NHANES data sample screening.

After data processing and filtering of NHANES data, a total of 13,902 valid samples were obtained, including 627 gout cases and 13,275 controls (Figure 7). The baseline study data indicated that magnesium intake in the gout patient group was significantly lower than that in the control group (*p* < 0.001). Additionally, significant differences were found in factors such as age, sex, BMI, smoking status, high blood pressure, high cholesterol level, and diabetes between the gout group and the control group (Table 3).

**Table 3:** All information of NHANES database in this study.

Characteristics	Overall	No	Yes	*P*-value
N	13,902	13,275	627	
Dietary intake magnesium	0.294 (0.135)	0.295 (0.136)	0.274 (0.126)	**<0.001**
Age	49.187 (17.713)	48.537 (17.645)	62.952 (12.892)	**<0.001**
BMI	29.284 (7.051)	29.161 (7.004)	31.889 (7.519)	**<0.001**
Gender (%)				
Female	7,041 (50.6%)	6,832 (51.5%)	209 (33.3%)	**<0.001**
Male	6,861 (49.4%)	6,443 (48.5%)	418 (66.7%)	
Alcohol (%)				
No	3,954 (28.4%)	3,795 (28.6%)	159 (25.4%)	0.088
Yes	9,948 (71.6%)	9,480 (71.4%)	468 (74.6%)	
Smoke (%)				
No	7,855 (56.5%)	7,585 (57.1%)	270 (43.1%)	**<0.001**
Yes	6,047 (43.5%)	5,690 (42.9%)	357 (56.9%)	
High Blood Pressure (%)				
No	8,764 (63.0)	8,602 (64.8)	162 (25.8)	**<0.001**
Yes	5,138 (37.0)	4,673 (35.2)	465 (74.2)	
High Cholesterol Level (%)				
No	8,989 (64.7)	8,734 (65.8)	255 (40.7)	**<0.001**
Yes	4,913 (35.3)	4,541 (34.2)	372 (59.3)	
Diabetes (%)				
No	11,672 (84.0)	11,283 (85.0)	389 (62.0)	**<0.001**
Yes	2,230 (16.0)	1992 (15.0)	238 (38.0)	

The bold values indicate statistical significance (*P* < 0.05).

#### 3.3.2 Dietary magnesium intake and logistic regression analysis

Dietary magnesium intake was categorized into quartiles: Q1 < 0.203 g, Q2 = 0.203 to <0.272 g, Q3 = 0.272 to <0.358 g, and Q4 ≥ 0.358 g. In the univariate logistic regression model (model 1), high magnesium intake (Q4) was significantly associated with lower gout risk than low magnesium intake (Q1) (OR = 0.788, 95% CI: 0.628–0.990, *p* = 0.046). After adjustment for sex and age as covariates in the multivariate model (model 2), magnesium intake remained a protective factor (OR = 0.685, 95% CI: 0.521–0.899, *p* = 0.009). Finally, in the fully adjusted model (model 3), incorporating all covariates, high magnesium intake (Q4) continued to demonstrate a protective effect against gout (OR = 0.738, 95% CI: 0.550–0.989, *p* = 0.049) (Table 4; Supplementary Table 8). This result has borderline statistical significance, indicating that dietary magnesium intake is possibly associated with gout risk and may have potential protective effects.

**Table 4:** The logistic regression model of exposure factor in NHANES database.

Dietary intake magnesium	Gout		Model 1				Model 2				Model 3			
	Yes	No	*OR*	95%*CI* low	95%*CI* up	*P*-value	*OR*	95%*CI* low	95%*CI* up	*P*-value	*OR*	95%*CI* low	95%*CI* up	*P*-value
Q1: <0.203 g	190	3,297	Reference											
Q2: 0.203 to <0.272 g	161	3,314	0.987	0.772	1.261	0.915	0.920	0.715	1.185	0.523	0.918	0.711	1.185	0.514
Q3: 0.272 to <0.358 g	146	3,324	0.892	0.640	1.243	0.504	0.797	0.563	1.129	0.209	0.820	0.580	1.159	0.269
Q4: ≥0.358 g	130	3,340	0.788	0.628	0.990	**0.046**	0.685	0.521	0.899	**0.009**	0.738	0.550	0.989	**0.049**

The bold values indicate statistical significance (*P* < 0.05).

#### 3.3.3 Subgroup analysis of the association between dietary magnesium intake and gout

**Figure 8:**
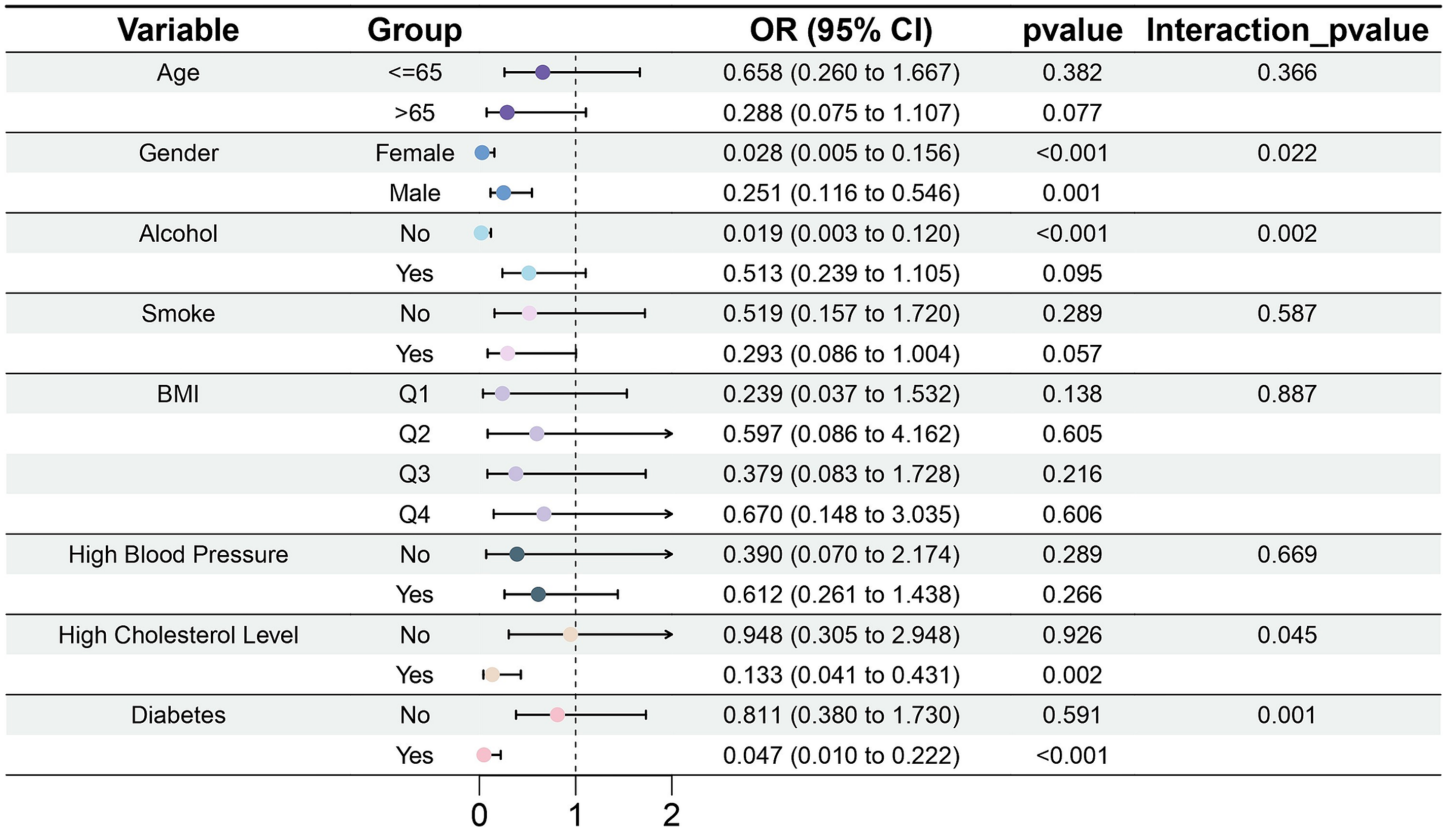
Forest plot of subgroup analysis of the effect of dietary magnesium intake on gout.

Magnesium intake did not significantly differ among groups stratified by age, smoking status, and BMI (Figure 8). Among participants who did not drink alcohol, magnesium intake was associated with diminished risk of gout (OR = 0.019, 95% CI: 0.003–0.120, *p* < 0.001). In the subgroup with high cholesterol level (OR = 0.133, 95% CI: 0.041–0.431, *p* = 0.002) and diabetes (OR = 0.047, 95% CI: 0.010–0.222, *p* < 0.001), the association between magnesium intake and gout was statistically significant. Moreover, we observed interactions of sex, alcohol consumption, high cholesterol level, and diabetes with magnesium intake (*p* < 0.05).

#### 3.3.4 Restricted cubic spline analysis

**Figure 9:**
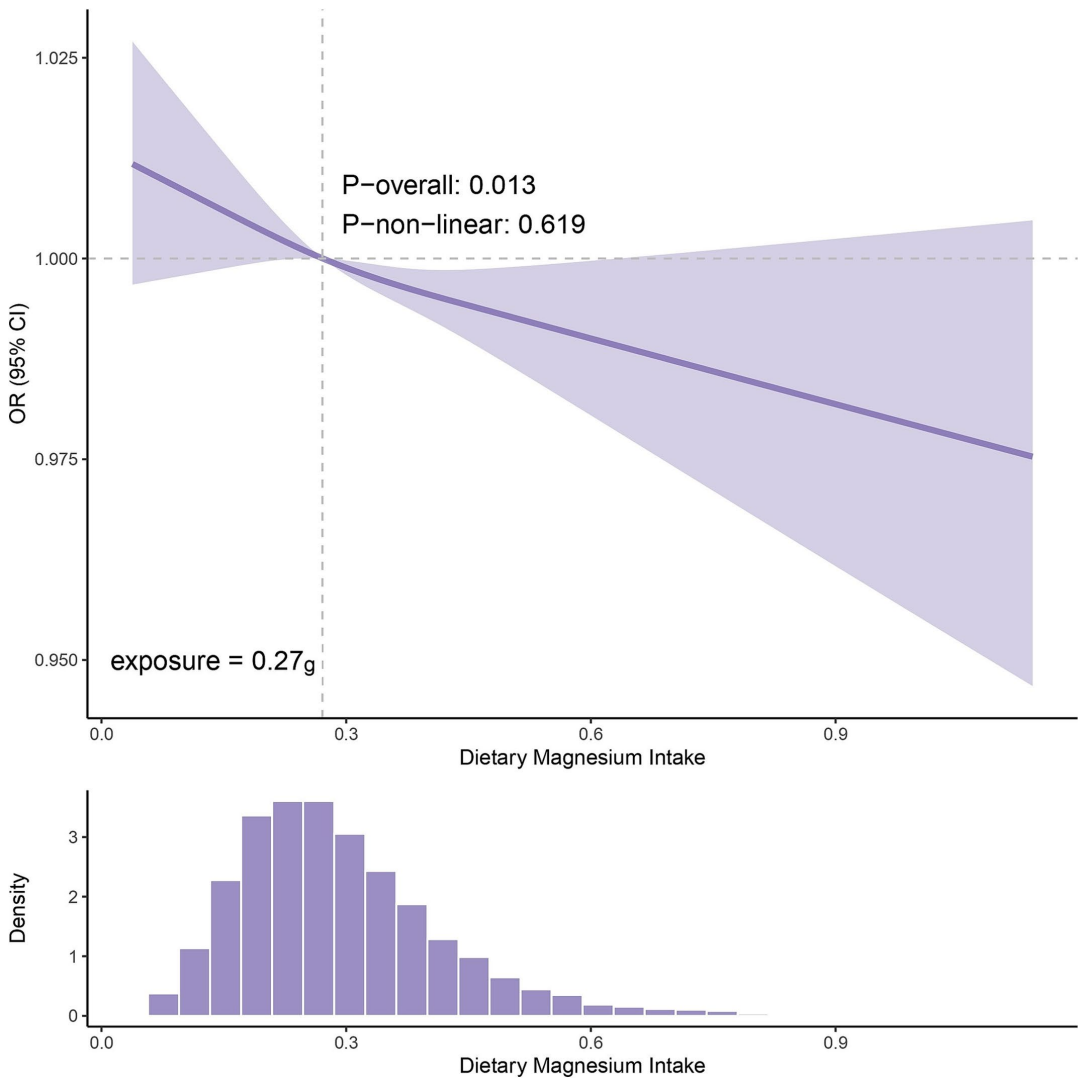
The curve of OR with dietary magnesium intake and the distribution histogram.

The RCS analysis of NHANES data revealed a dose–response relationship between dietary magnesium intake and gout risk (*p* for overall association = 0.013; *p* for nonlinearity = 0.619). As shown in Figure 9, the curve exhibited a generally monotonic downward trend, indicating that the risk of gout gradually decreased with increasing magnesium intake. The inflection point of the curve was observed at an intake level of approximately 0.27 g/day, beyond which the OR consistently remained below 1. This suggests a potential threshold effect, whereby dietary magnesium intake above this level may be associated with a reduced risk of gout. Further clinical trials are required to validate these findings.

## 4 Discussion

This study integrated MR analysis with cross-sectional epidemiological data from China and the United States to validate the relationship between magnesium and gout through multilevel approaches. First, we identified magnesium, a micronutrient strongly associated with gout, through MR analysis. We then validated the association between serum magnesium and gout by using clinical data from China. Additionally, by analyzing the NHANES database, we explored the relationship between dietary magnesium intake and gout risk. Our findings suggested that magnesium might have a protective role against gout, on the basis of a negative correlation between dietary magnesium intake and gout risk.

### 4.1 Evidence of the association between magnesium and gout risk

Magnesium, a critical trace element stored predominantly in bone tissue with minimal circulating levels (17), serves as a cofactor and activator for more than 300 enzymatic reactions (18). This micronutrient plays critical roles in energy metabolism, protein synthesis, muscle contraction, and neurotransmission (19). Magnesium deficiency has been associated with the pathogenesis of chronic diseases, including metabolic disorders (20). Both gout and HUA arise from impaired purine metabolism, and emerging evidence highlights magnesium’s regulatory role in this context. A cross-sectional study in 5,168 Chinese participants has revealed an inverse association between dietary magnesium intake and HUA risk in males, while this effect was not observed in females (21). Similarly, NHANES data have corroborated the correlation between higher magnesium and lower HUA risk (22), whereas magnesium deficiency has shown positive associations with HUA prevalence (23) and gout incidence (24). In addition, other studies have reported that high magnesium intake was linearly correlated with all-cause mortality in patients with gout and HUA, and demonstrated an association between higher magnesium intake and lower risk of all-cause mortality (25).

### 4.2 Potential biological mechanisms linking magnesium and gout risk

The biological mechanism between magnesium and gout risk has not been fully elucidated, but it may be related to the inflammatory process. Emerging evidence indicates a significant inverse correlation between serum magnesium levels and high-sensitivity C-reactive protein (hs-CRP) concentrations (26, 27), and hypomagnesemia is independently associated with elevated CRP (28). Notably, magnesium supplementation has been shown to effectively attenuate CRP levels (29), thus suggesting a potential modulatory role of magnesium in inflammatory processes. Additionally, elevated CRP levels correlate with elevated all-cause and cancer-specific mortality in patients with gout (30). Studies have found that CRP is closely related to NLRP3. Activation of NLRP3 inflammasomes plays a critical role in gout pathogenesis (31, 32). Beyond enhancing NLRP3 expression through FcγRs/NF-κB signaling (33), CRP promotes diabetic nephropathy progression through Smad3-mediated activation of NLRP3 inflammasomes (34). Furthermore, magnesium may influence gout risk by modulating uric acid production pathways. As an essential cofactor for DNA and RNA synthesis, magnesium plays a critical role in maintaining nucleic acid integrity (35). Research suggests that magnesium deficiency can compromise DNA stability and repair efficiency, potentially leading to aberrant purine nucleotide degradation and consequently increasing endogenous uric acid generation (23, 36). In summary, magnesium might play a protective role in gout by modulating inflammation and uric acid levels. Its precise mechanisms require further experimental validation. We hypothesize that moderately increasing dietary magnesium intake may help control inflammatory responses and decrease uric acid levels, thereby potentially reducing the risk of gout.

### 4.3 Influences of subgroup factors on the magnesium-gout association

Uric acid is excreted primarily via the kidneys. Renal dysfunction, as occurs in CKD, AKI, NS, and GN, impairs uric acid excretion capacity, thereby increasing the risk of gout development (37, 38). Our clinical data further demonstrated that serum magnesium might protect against gout risk in patients with CKD. Although gout shows a male predominance (39), our data unexpectedly indicated an association between higher serum magnesium and greater gout risk in females, a finding potentially attributable to limited female cases in our study (Control: 1805; Case: 10). Therefore, sex-specific magnesium homeostasis mechanisms require further investigation. Our study additionally identified significant interactions of magnesium intake with alcohol consumption, hyperlipidemia, and diabetes status, in agreement with previous reports (40–44). In summary, our findings underscore the roles of lifestyle factors, diverse metabolic disorders, and renal diseases in the pathogenesis of gout. We suggest implementing a comprehensive multifactorial management strategy in clinical practice to more effectively prevent and manage gout.

### 4.4 Significance and assessment of dietary magnesium intake in patients with gout

According to the Chinese dietary reference intakes, the recommended daily magnesium intake is 330 mg for adults, and an additional 40 mg is required for pregnant and lactating people. These values closely align with the U.S. recommended dietary allowance of 310–420 mg/day (45). Currently, the dietary magnesium intake in both Western and Eastern countries is substantially below recommended daily levels and indicates widespread deficiency (46, 47). Therefore, increased dietary intake of magnesium-rich foods, primarily leafy green vegetables (e.g., spinach), fruits (e.g., bananas), whole grains (e.g., brown rice and oats), and nuts (e.g., cashews and almonds), is recommended (48). Furthermore, appropriate cooking methods should be used to minimize magnesium loss during food preparation (46). Additionally, under physician supervision, magnesium supplementation (e.g., magnesium sulfate or magnesium chloride) might be considered to address potential deficiencies (49, 50). From a translational perspective, this study, based on NHANES data, revealed a significant association between a dietary magnesium intake exceeding 0.27 g and diminished gout risk (OR <1), thus providing empirical support for defining intervention thresholds. Our findings suggested that increasing dietary magnesium intake might be associated with a reduced risk of gout. This finding requires further validation through subsequent clinical trials or prospective cohort studies.

### 4.5 Limitations

Herein, we not only conducted MR analysis on European cohort data but also integrated Chinese clinical data and U.S. NHANES data, thereby enhancing the generalizability of the findings. However, several limitations should be acknowledged, as follows. (1) Lack of assessment of magnesium storage status: the MR methods and clinical data used in this study reflected only circulating magnesium levels but not storage status. (2) Inclusion bias of study participants: although the sample size exceeded the epidemiological calculation thresholds, the underrepresentation of female patients with gout might potentially introduce bias. Additionally, because all collected clinical data came from patients with kidney disease, a control group of healthy individuals was lacking. Moreover, because of data collection and organization constraints, the temporal baseline in the Chinese clinical data differed from that in NHANES. (3) Observational analysis error: Dietary magnesium intake data are derived from the 24-h recall method, and inherent measurement errors in this method may affect the accuracy of the assessment. In the NHANES data, gout outcomes rely on patient self-reporting, and such misclassification may introduce bias into the observed associations. Residual confounding is likely present in Chinese clinical data, such as drug use (diuretics, allopurinol, colchicine, steroids, PPIs, etc.), socioeconomic status, and total energy and protein intake. (4) Multiple Testing Issue: To comprehensively explore potential associations between exposure to 15 nutrients and gout, this study did not perform multiple testing correction during the preliminary analysis phase. While this strategy aids in identifying potential factors, it correspondingly increases the risk of false positive results. (5) Lack of intervention experiments: a prior study has reported a positive correlation between magnesium deficiency and gout risk in U.S. adults, but dietary magnesium intake did not modify this association (24). In Model 3 of NHANES in this study, dietary magnesium intake had a potential protective effect on gout (*p* = 0.049), which should be interpreted with caution. Therefore, the efficacy of magnesium supplementation requires further validation through randomized controlled trials. (6) Lack of mechanistic validation: This study relied on a literature-based synthesis to formulate hypotheses regarding the potential mechanisms of magnesium in gout pathophysiology. However, the specific mechanisms will require further experimental verification. In summary, despite this study’s advancements in elucidating the relationship between magnesium and gout, further exploration remains necessary to address existing limitations. Future research should prioritize expanding the study sample, incorporating healthy individuals, and providing more robust experimental evidence to strengthen the findings.

## 5 Conclusion

Our study integrated Mendelian randomization analysis, clinical data validation, and dietary intake assessment across diverse populations. The results indicate that higher magnesium intake may reduce the risk of gout. These findings support the development of gout prevention strategies and dietary interventions. Future randomized controlled trials are required to elucidate the underlying mechanisms linking magnesium intake to gout.

## Data Availability

The original contributions presented in the study are included in the article/Supplementary material, further inquiries can be directed to the corresponding authors.

## Footnotes

^1^ https://www.finngen.fi/en

## Supplementary material

Supplementary Figure 1 MR Analysis Forest Plot of other micronutrients.

Supplementary Figure 2 Forest plots of leave-one-out sensitivity analysis of other micronutrients.

Supplementary Figure 3 Scatter plots of other micronutrients.

Supplementary Figure 4 Funnel plots of other micronutrients.

Supplementary Figure 5 Violin plot of eGFR distribution across different serum magnesium levels.

Supplementary Material 1 The main code of the MR analysis is in Supplementary Material, and other statistical analysis codes can be obtained by contacting the corresponding author.
