# Supplementary figures and images for: The association between magnesium levels and gout: evidence from Mendelian randomization, a Chinese cross-sectional study, and NHANES analysis
Source: Front Nutr. 2025 Nov 25;12:1688095. doi: 10.3389/fnut.2025.1688095 (PMC12687734; doi:10.3389/fnut.2025.1688095)

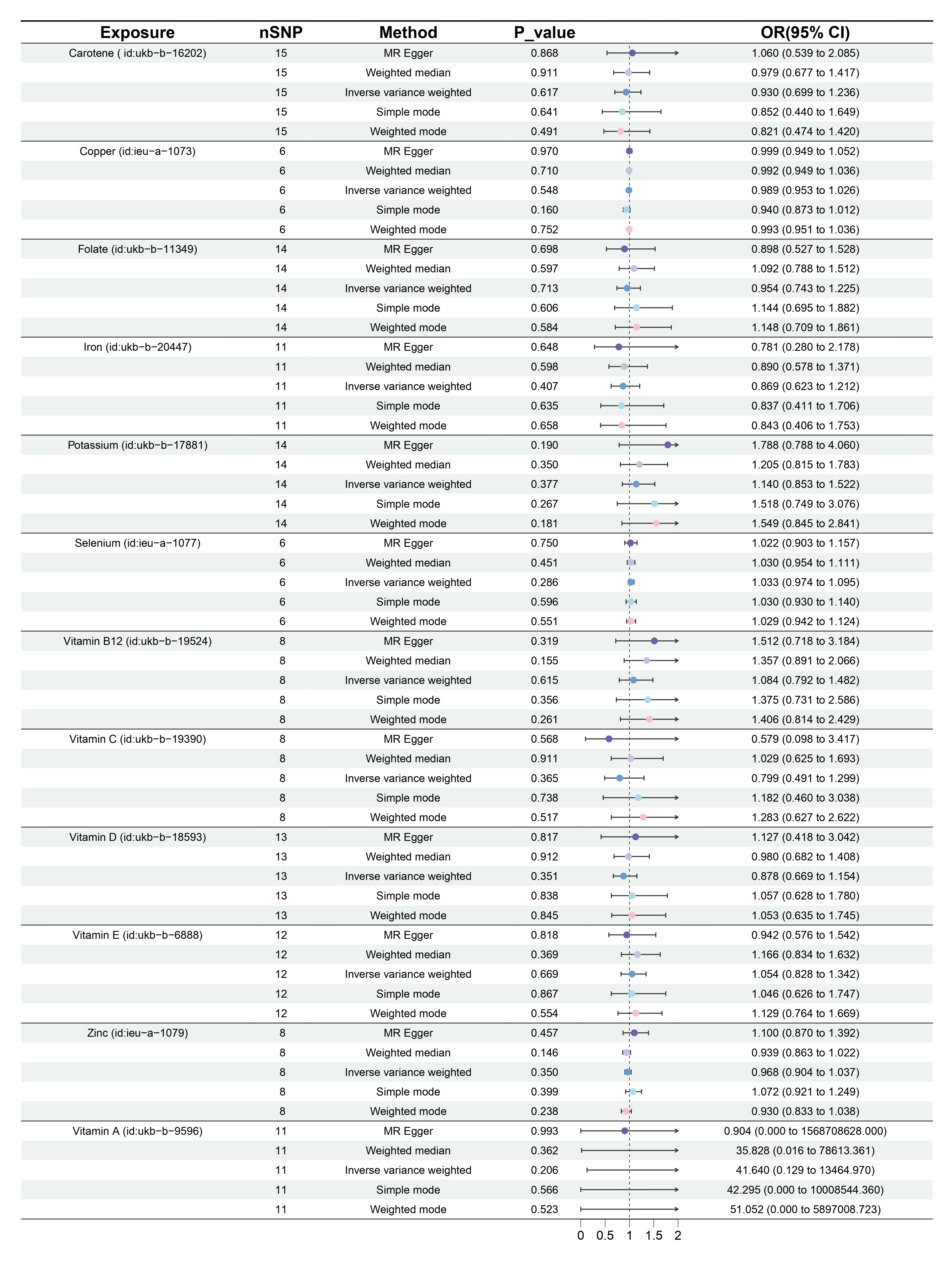

Supplement: Supplementary file 1 [file Image_1.jpeg]

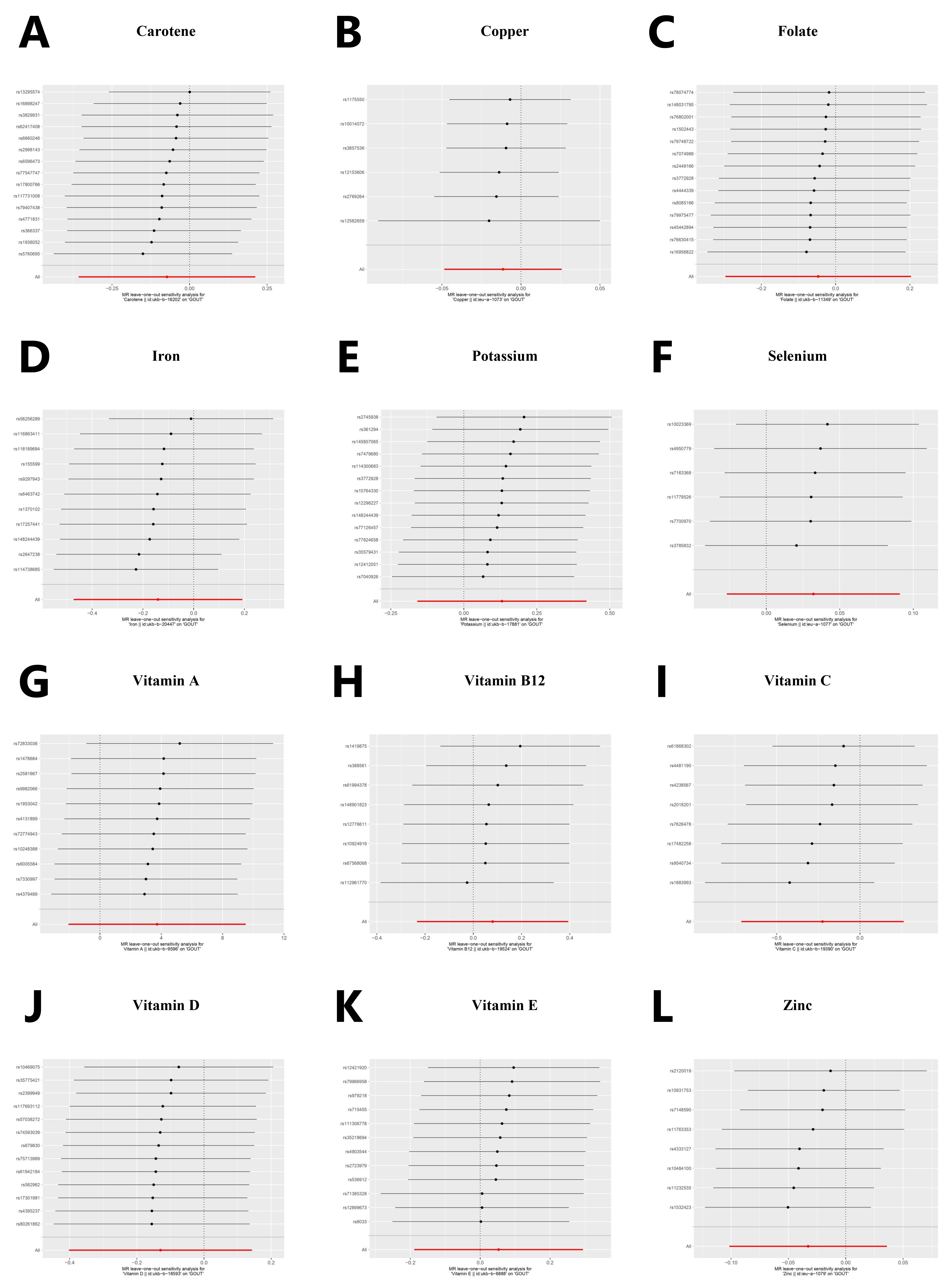

Supplement: Supplementary file 2 [file Image_2.jpeg]

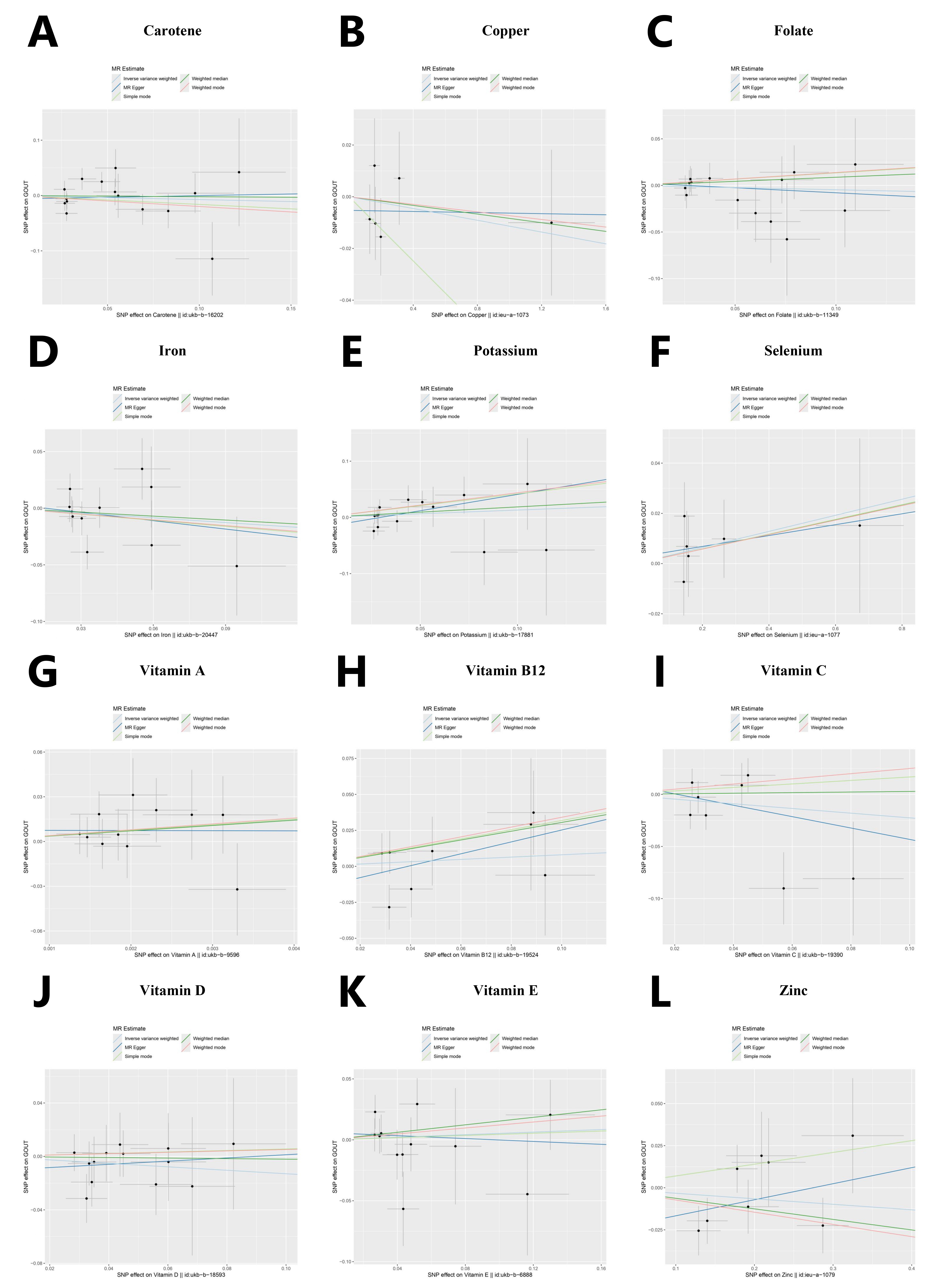

Supplement: Supplementary file 3 [file Image_3.JPEG]

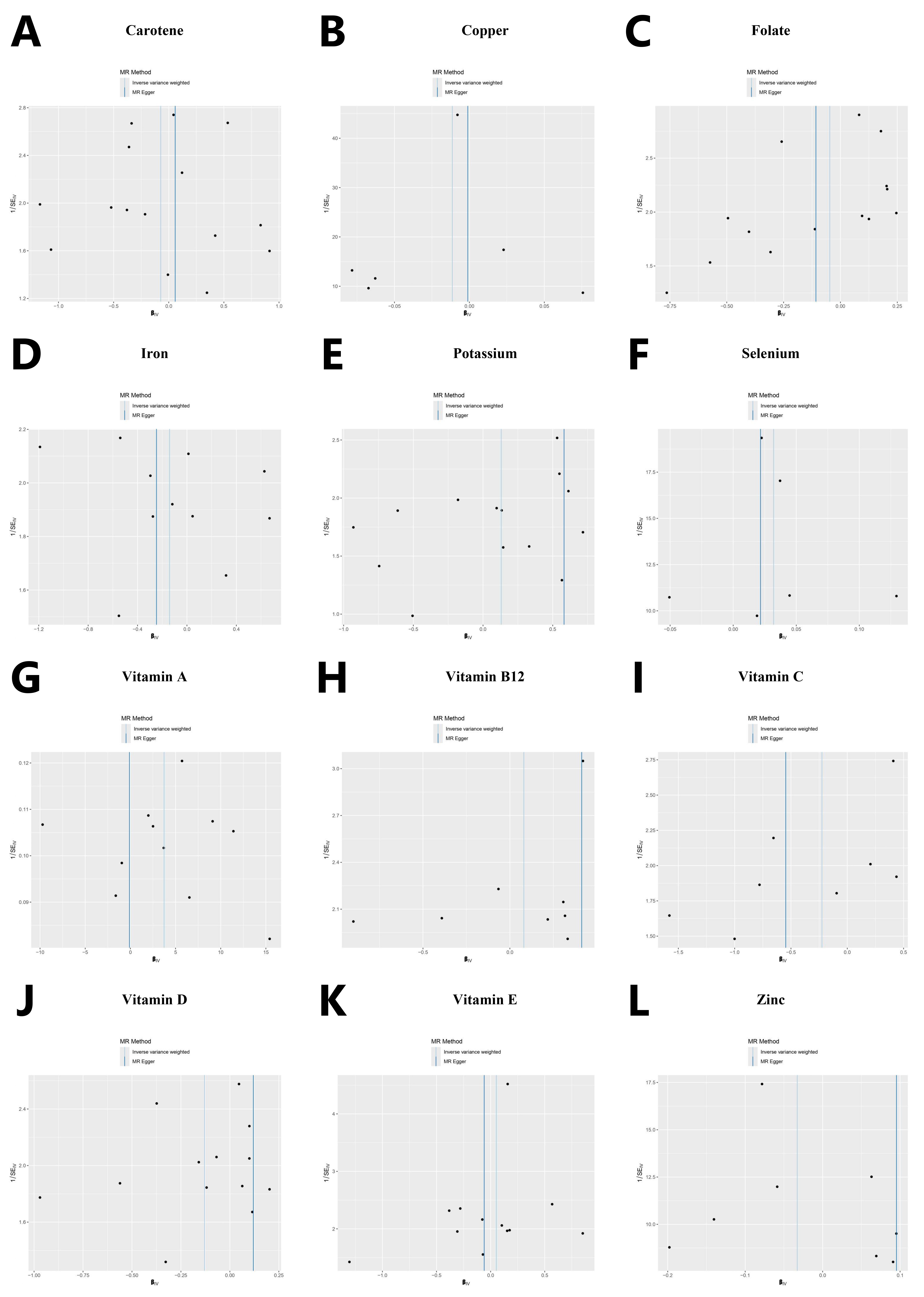

Supplement: Supplementary file 4 [file Image_4.JPEG]

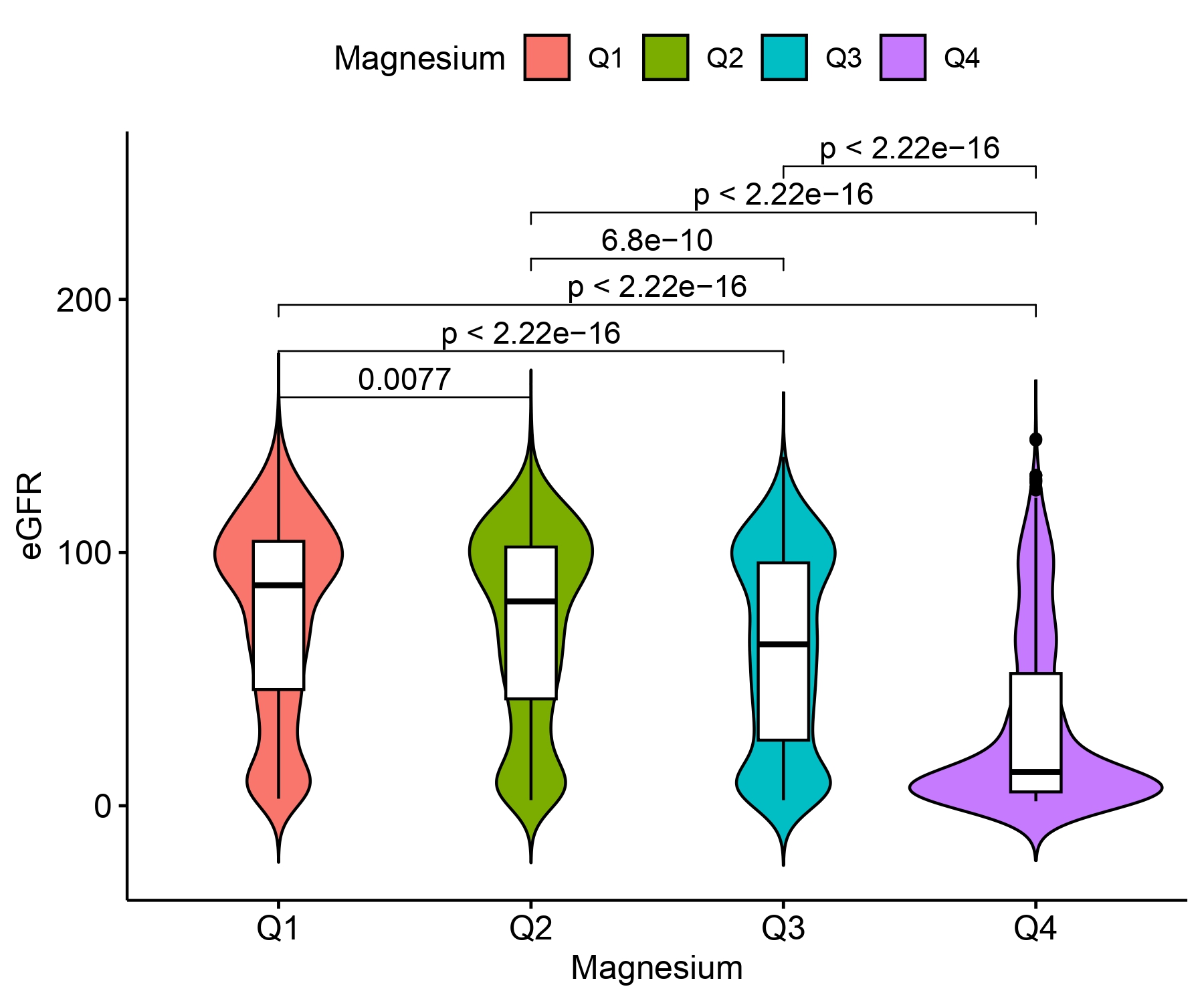

Supplement: Supplementary file 5 [file Image_5.JPEG]
