# Supplementary material for: The association between magnesium levels and gout: evidence from Mendelian randomization, a Chinese cross-sectional study, and NHANES analysis
Source: Front Nutr. 2025 Nov 25;12:1688095. doi: 10.3389/fnut.2025.1688095 (PMC12687734; doi:10.3389/fnut.2025.1688095)
Supplement: Supplementary file 6 [file Supplementary_file_1.docx]

library(VariantAnnotation)

library(TwoSampleMR)

exposureFile="exposure.F.csv"

outcomeFile="finngen_R12_M13_GOUT.gz"

outcomeName="GOUT"

exposure_dat=read_exposure_data(filename=exposureFile,

sep = ",",

snp_col = "SNP",

beta_col = "beta.exposure",

se_col = "se.exposure",

pval_col = "pval.exposure",

effect_allele_col="effect_allele.exposure",

other_allele_col = "other_allele.exposure",

eaf_col = "eaf.exposure",

id_col = "id.exposure",

phenotype_col = "exposure",

samplesize_col = "samplesize.exposure",

chr_col="chr.exposure", pos_col = "pos.exposure",

clump=FALSE)

outcomeData=read_outcome_data(snps=exposure_dat$SNP,

filename=outcomeFile, sep = "\t",

snp_col = "rsids",

beta_col = "beta",

se_col = "sebeta",

effect_allele_col = "alt",

other_allele_col = "ref",

pval_col = "pval",

eaf_col = "af_alt")

write.csv(outcomeData, file="outcome.csv", row.names=F)

outcomeData$outcome=outcomeName

dat=harmonise_data(exposure_dat, outcomeData)

presso <- run_mr_presso(dat, NbDistribution = 1000)

outTab=dat[dat$mr_keep=="TRUE",]

write.csv(outTab, file="table.SNP.csv", row.names=F)

mrResult=mr(dat)

mrTab=generate_odds_ratios(mrResult)

write.csv(mrTab, file="table.MRresult.csv", row.names=F)

heterTab=mr_heterogeneity(dat)

write.csv(heterTab, file="table.heterogeneity.csv", row.names=F)

pleioTab=mr_pleiotropy_test(dat)

write.csv(pleioTab, file="table.pleiotropy.csv", row.names=F)

pdf(file="pic.scatter_plot.pdf", width=7.5, height=7)

mr_scatter_plot(mrResult, dat)

dev.off()

res_single=mr_singlesnp(dat)

pdf(file="pic.forest.pdf", width=7, height=5.5)

mr_forest_plot(res_single)

dev.off()

pdf(file="pic.funnel_plot.pdf", width=7, height=6.5)

mr_funnel_plot(singlesnp_results = res_single)

dev.off()

pdf(file="pic.leaveoneout.pdf", width=7, height=5.5)

mr_leaveoneout_plot(leaveoneout_results = mr_leaveoneout(dat))

dev.off()
